# COMPARING COVID-19 CASES AND MORTALITY RATES: THE GREEN HOUSE VERSUS THE TRADITIONAL NURSING HOME IN THE UNITED STATES

**DOI:** 10.1093/geroni/igad104.1143

**Published:** 2023-12-21

**Authors:** Yuchi Young, Virgile Barnes, Taylor Perre, Wan-Hsiang Hsu, Ashley Shayya, Thomas O’Grady

**Affiliations:** University at Albany, Rensselaer, New York, United States; SUNY Albany, School of Public Health, Albany, New York, United States; Home Care Association of New York State, Albany, New York, United States; University at Albany, Albany, New York, United States; University at Albany School of Public Health, Rensselaer, New York, United States; State University of New York at Albany, Albany, New York, United States

## Abstract

**Introduction:**

The COVID-19 pandemic has posed significant challenges to nursing home (NH) care. However, Green Houses (GH), with their small size and unique care model, may offer a solution for infection control and COVID-19 prevention. This study aims to compare COVID-19 case and mortality rates between GHs and traditional NHs in the United States.

**Methods:**

Secondary data were used to compare COVID-19 case and mortality rates among three groups: GHs (n=19), traditional small NHs (n=266), and traditional large NHs (n=2932) located in 10 US states from June 2020 to September 2022. Poisson regressions using generalized estimating equations (GEE) were utilized to quantify differences in COVID-19 case and mortality rates while controlling for average age, gender, and ADL score.

**Results:**

The study population had a mean age of 73.38 and was primarily female (57.8%) and white (78.2%). The GEE results indicated that small and large NHs have significantly higher relative risks (RR) of COVID-19 cases (RR = 1.61; 95% CI 1.25-2.08 and RR = 1.75; 95% CI 1.36-2.24, respectively) compared to GHs. For COVID-19 mortality rates, there is an increased risk in large NHs compared to GHs (RR = 1.67; 95% CI 1.01-2.77), but no difference between GHs and small NHs.

**Conclusion:**

After adjusting for age, gender, and ADL disability, GHs have lower COVID-19 incidence and mortality rates than traditional NHs. These differences may be attributable to GHs having fewer beds, offering more resident privacy, and having a higher certified nursing assistant-to-resident ratio compared to traditional NHs.

